# Blood parameters associated with residual feed intake in beef heifers

**DOI:** 10.1186/s13104-023-06444-6

**Published:** 2023-08-18

**Authors:** Brooke A. Clemmons, Taylor B. Ault-Seay, Madison T. Henniger, M. Gabbi Martin, Pierre-Yves Mulon, David E. Anderson, Brynn H. Voy, Kyle J. McLean, Phillip R. Myer

**Affiliations:** 1 Department of Agriculture, College of Agricultural Sciences and Natural Resources, Texas A&M University – Commerce, Commerce, TX USA; 2 Department of Animal Science, University of Tennessee Institute of Agriculture, University of Tennessee, Knoxville, TN USA; 3 Department of Large Animal Clinical Sciences, College of Veterinary Medicine, University of Tennessee, Knoxville, TN USA

**Keywords:** Cattle, Blood, Feed efficiency

## Abstract

Blood chemistry may provide indicators to greater feed efficient cattle. As a side objective to previous research, 17 Angus heifers approximately two years old underwent a feed efficiency trial to determine residual feed intake (RFI) and identify variation in blood chemistry in beef cattle divergent in feed efficiency. Heifers were categorized as high- or low-RFI based ± 0.25 standard deviations around mean RFI. Blood samples were analyzed using an i-STAT handheld blood analyzer to measure sodium, potassium, glucose, blood urea nitrogen (BUN), creatinine, hematocrit, and hemoglobin. BUN was greater in high-RFI heifers (µ = 8.7 mg/dL) contrasted to low-RFI heifers (µ = 6.5 mg/dL; *P* = 0.01), whereas glucose was greater in low-RFI heifers (µ = 78.1 mg/dL) contrasted to high-RFI heifers (µ = 82.0 mg/dL; *P* = 0.05). No other blood chemistry parameters differed by RFI. The greater abundance of BUN in high-RFI heifers may indicate inefficient utilization of protein or mobilization of tissue protein for non-protein use. Greater blood glucose concentrations in low-RFI heifers may indicate greater utilization of energy precursors, such as volatile fatty acids, or metabolites. These data suggest there are readily measurable indicators of physiological variation in nutrient utilization; however, this warrants additional studies to explore.

## Introduction

The beef cattle industry accounts for billions of dollars in gross domestic product for the United States and feed accounts for approximately 40–60% of production costs [1]. Improving feed efficiency in beef cattle stands to increase productivity while simultaneously decreasing input resources, as well as decreased land use for animal agriculture [2]. Although the benefits of improving feed efficiency are well-demonstrated, feed efficiency is a complex, composite phenotype resulting from various dynamic underlying components. Identifying and determining the impact of the underlying components of feed efficiency is critical to facilitate the development of predictors of feed efficiency.

The overall objective of this study was to evaluate the rumen microbiome, rumen metabolome, and blood metabolome in beef heifers to identify biomarkers of feed efficiency [3]. The current research presented is a component of another study in which ruminal bacterial communities and metabolome variation were identified in beef heifers divergent in feed efficiency. In this portion of the study, the objective was to determine blood chemistry in heifers that varied in residual feed intake (RFI).

## Methods

### Experimental design and sample collections

In this study, 17 previously cannulated Angus heifers sourced from the University of Tennessee were used that were approximately two years of age and weighed 563 ± 12 kg at the beginning of the study. The heifers were housed at the University of Tennessee Plateau Research and Education Center in Crossville, TN prior to the initiation of the study. To determine RFI, individual feed intake was monitored using the GrowSafe automated feeding system (Model 6000, GrowSafe Systems Ltd., Airdrie, Alberta, Canada) with a primarily corn silage-based ration [3]. Prior to the initiation of the study, heifers were allowed a two-week adaptation period to acclimate to the diet and GrowSafe system. Heifers then underwent a 70-day feed efficiency trial [3]. Body weights were measured on days − 1, 1, 35, 69, and 70 of the 70-day feed efficiency trial. Average daily feed intake and RFI calculations were determined using the GrowSafe system. Residual feed intake was calculated for each heifer based on previously established methods [4]. On day 70 of the feed efficiency trial and prior to daily feeding, blood was collected via coccygeal venipuncture into a 5 mL lithium heparin tube for serum analyses. After blood collection, lithium heparin tubes were inverted gently five times to mix the sample with the contents of the tube and then immediately analyzed using the i-STAT CHEM8 + cartridge on the i-STAT 1 handheld system (Abbott Laboratories, Chicago, IL, USA) according to manufacturer instructions. The i-STAT CHEM8 + measured sodium (Na), potassium (K), chloride (Cl), total blood carbon dioxide (TCO_2_), anion gap, ionized calcium (iCa), glucose (Glu), urea nitrogen/urea ratio (BUN/UREA), creatinine (Crea), hematocrit (Hct), and hemoglobin (Hgb).

### Statistical analyses

Data were analyzed using SAS 9.4 (SAS, Cary, NC, USA). The distribution of the residuals of the data (Na, K, Cl, TCO_2_, anion gap, Glu, BUN/UREA, Crea, Hct, and Hgb) were assessed for normality. Ionized calcium was excluded from analyses due to missing and unreadable data points. Data were considered normally distributed based on visual assessment of histograms and a Shapiro-Wilk value of ≥ 0.80 and all data followed a normal distribution. The blood chemistry values were then analyzed using a mixed-model ANOVA with fixed effect of RFI designation and random effect of pen. Statistical differences were determined at p ≤ 0.05.

## Results

Heifers were classified as high- or low-RFI heifers based on ± 0.25 standard deviations about the mean RFI [3]. Six heifers were classified as high-RFI (n = 6), and eight heifers were classified as low-RFI (n = 8). Differences between high- and low-RFI heifers were not observed in Na, K, Crea, Hct, and Hgb (*P* > 0.05; Table 1). However, BUN/UREA was greater in the blood of high-RFI heifers (µ = 8.7 mg/dL) contrasted to low-RFI heifers (µ = 6.5 mg/dL; *P* = 0.01; Table 1). Blood Glu was greater in low-RFI heifers (µ = 78.1 mg/dL) contrasted to high-RFI heifers (µ = 82.0 mg/dL; *P* = 0.05; Table 1).

**Table 1:** Blood chemistry/electrolytes in high- and low-RFI beef heifers

Metabolite	High RFI^a^	Low RFI^a^	*P*-Value^b^
Sodium^c^	138.14 ± 0.68	137.88 ± 0.63	0.78
Potassium^c^	3.94 ± 0.18	3.85 ± 0.17	0.72
Glucose^d^	78.57 ± 1.30	82.00 ± 1.22	0.05
BUN/Urea^d^	8.71 ± 0.57	6.05 ± 0.53	0.01
Creatinine^d^	1.01 ± 0.05	1.08 ± 0.05	0.42
Hematocrit^e^	29.57 ± 0.66	28.63 ± 0.62	0.31
Hemoglobin^f^	10.06 ± 0.23	9.73 ± 0.21	0.31

^a^ Mean ± SEM

^b^ Statistical differences were determined at p ≤ 0.05

^c^ mmol/L

^d^ mg/dL

^e^ (%)

^f^ g/dL

## Discussion

Feed efficiency in food animal production is a highly sought-after trait to maximize outputs from costly feed inputs [4]. While phenotypic evaluation is a valuable tool in the genetic selection process, phenotypic evaluation for actual performance is just as critical. Residual feed intake is a feed efficiency metric that determines the difference between the actual dry matter intake and the expected dry matter intake of an animal required for maintenance and growth. This is estimated through a regression equation involving metabolic body weight and average daily gain [4]. Although RFI is an often-used measurement, especially in research, feed trials often are difficult to perform due to costly equipment, labor, and time requirements.

Studies have attempted to use other biological data, such as metabolites in blood, to correlate to feed efficiency with the rationale to eventually use such data as indicators of feed efficiency without necessitating the lengthy or expensive feeding trials required for this phenotype. Kelly et al. (2010) examined the relationship between feed efficiency and plasma metabolites in finishing beef heifers [5]. The authors used RFI as a measure of feed efficiency and several plasma metabolites, including urea, β-hydroxybutyrate, and insulin, were correlated with RFI. However, in the study conducted by Kelly et al., glucose and urea did not differ by RFI phenotype and has been observed in other studies [6, 7]. This contrasts with the present study in which we observed differences by feed efficiency phenotype in blood glucose, with lower concentrations of glucose observed in high-RFI heifers contrasted to low-RFI heifers. These conflicting data could be observed due to differences in equipment used, breed and age of heifers, and diet. Given the importance of glucose as an energy metabolite, glucose may be of use as a marker of feed efficiency in some heifers.

Glucose is vitally important as the primary source of energy used by most animals [8]. In ruminants, the main source of glucose results from gluconeogenesis using volatile fatty acids (VFAs), namely propionate [9] derived from the ruminal microbiota [10]. Due to continuous ruminal fermentation, ruminants do not experience the greater fluctuations in blood glucose concentrations following meals as non-ruminants, although it does occur to a lesser extent. Regardless of the lesser fluctuations, glucose regulation and availability remain important factors in overall health and production of ruminant species [8, 9].

In the current study, the BUN:Urea ratios differed by RFI. Blood urea nitrogen is frequently used as a relatively easy method for assessing protein status in ruminants [11]. Blood urea nitrogen is also associated with reproductive performance in heifers. In a study conducted by Tshuma et al. in 2014, the authors examined the concentration of BUN in pre-breeding heifers [12]. The researchers observed that heifers with greater pre-breeding BUN took longer to become pregnant than those with lower quantities [12]. Additionally, BUN is frequently used as an indicator of feed efficiency in beef cattle. In a meta-analysis conducted by Datt et al. (2017), the authors reported a positive correlation between RFI and BUN, with an ‘r’ value of 0.73, which was significant [13]. In the present study, high-RFI heifers had greater BUN concentrations than low-RFI heifers, and this may have multiple impacts on production. BUN or BUN:urea concentrations may serve as indicators of feed efficiency, primarily that greater BUN or BUN:urea concentrations suggest less feed efficient heifers, and potentially decreased reproductive performance.

## Limitations

There were several limitations to this study. Primarily, the low number of animals used in the study (n = 17) may have contributed to unobserved differences in the other metabolites measured. Further, in this study, the heifers were not maintained in a standard production manner. The heifers were previously cannulated and several months prior to the start of the study had been involved in multiple nutritional studies. These factors may have impacted the results observed.

## Data Availability

The datasets generated and/or analyzed during the current study are available in the FigShare repository, https://figshare.com/articles/dataset/Heifer_Feed_Efficiency_Blood_Parameters/22250926.

## Abbreviations

RFI: Residual feed intake
BUN: Blood urea nitrogen, Na = Sodium, K = Potassium, Glu = glucose
Crea: Creatinine
Hct: Hematocrit
Hgb: Hemoglobin
VFA: Volatile fatty acid
